# Physical Characterization of a Novel Carrot Juice Whey-Enriched Beverage Fermented with Milk or Water Kefir Starter Cultures

**DOI:** 10.3390/foods12183368

**Published:** 2023-09-08

**Authors:** Anita Rejdlová, Richardos Nikolaos Salek, Zuzana Míšková, Eva Lorencová, Vendula Kůrová, Richard Adámek, Daniela Sumczynski

**Affiliations:** 1 Department of Food Technology, Faculty of Technology, Tomas Bata University in Zlin, Nám. T. G. Masaryka 5555, 760 01 Zlin, Czech Republic; a_rejdlova@utb.cz (A.R.); miskova@utb.cz (Z.M.); lorencova@utb.cz (E.L.); v_kurova@utb.cz (V.K.); radamek@utb.cz (R.A.); 2 Department of Food Analysis and Chemistry, Faculty of Technology, Tomas Bata University in Zlin, Nám. T. G. Masaryka 5555, 760 01 Zlin, Czech Republic; sumczynski@utb.cz

**Keywords:** fermented beverage, carrot juice, whey, water kefir starter culture, milk kefir starter culture, physicochemical properties, sensory analysis

## Abstract

The purpose of this work was to evaluate the selected physicochemical, rheological, and sensory properties of a new whey-enriched carrot juice beverage (carrot juice: whey ratios of 100:0; 95:5; 85:15; 75:25; 65:35) fermented with milk or water kefir starter cultures over a storage period of 21 days (at 4 ± 1 °C). In general, for all tested samples, the values of total soluble solids, pH, and density decreased with increasing storage time. In contrast, the values of ethanol, degree of fermentation, and total dissolved solids increased with the prolongation of the storage time. Furthermore, it was found that all the model samples exhibited pseudoplastic behavior. Based on the sensory analysis performed, samples containing 25% (*w*/*w*) whey were evaluated as the most acceptable. Last but not least, the present study can serve as a basis for optimizing the manufacturing technology of a novel fermented vegetable beverage enriched with whey.

## 1. Introduction

In recent years, there has been increasing consumer interest in functional foods containing biologically active substances with possible positive effects on the human body [1]. In general, these types of products are described as functional foods. Additionally, the American Dietetic Association defines functional foods as “foods that are in the form of whole, fortified, enriched, or enhanced foods that provide functional advantages and/or health benefits beyond basic nutrition when consumed at an effective level on a regular basis” [2]. Functional foods include, for example, baby food, cereals, meat products, various spreads, dairy products, and beverages. In addition, even fermented products can be classified as functional foods [3]. Due to (i) consumer demands for container contents, size, shape, and appearance; (ii) ease of distribution and better storage for refrigerated and shelf-stable products; and (iii) numerous opportunities to include desirable nutrients and bioactive compounds, beverages are by far the most active functional food category [4,5].

Carrots (*Daucus carota*) contain compounds that have been experimentally shown to have anticarcinogenic and immunoactive properties, as well as the ability to maintain an appropriate level of blood sugar, cholesterol, and blood pressure [6]. Carrot juice is growing in popularity due to its balanced organoleptic and nutritional properties. The main benefits include a high content of carotenoids, a variety of vitamins (such as vitamins B1, B2, B6, B9, C, and K), fiber, and antioxidants [7,8].

Whey can be considered a suitable new ingredient for the production of fermented dairy beverages because lactic acid bacteria (LAB) can metabolize it [9,10]. Whey is produced as a by-product of the dairy industry. Furthermore, further use of whey could lead to a sustainable economy since up to 40% of whey is not processed, reducing environmental pollution. Whey is highly digestible and serves as a source of lactose, high-quality complete serum proteins, vitamins, and minerals (especially calcium, magnesium, and phosphorus) [11,12,13]. Furthermore, whey can provide the human body with positive effects, including antioxidant activity, antihypertensive, antidiabetic, or antimicrobial properties, and is therefore considered a suitable ingredient for the production of functional foods [13,14].

Moreover, kefir starter cultures can be used in the production of fermented beverages combined with fruit or vegetable components. Kefir is a frequently consumed beverage, especially in Eastern European and Central Asian countries, and contains significant amounts of protein, prebiotics, and probiotics [15]. The milk kefir grains are small, round, and white and represent a symbiotic microbial ecosystem composed of lactic acid bacteria (LAB), yeast, and a small amount of acetic acid bacteria (AAB) distributed in an exopolysaccharide complex of kefiran. The genera of microorganisms found in milk kefir starter culture include, for example, the genera *Lactobacillus*, *Lactococcus*, *Leuconostoc*, *Sacharomyces*, and *Kluyveromyces* [16,17,18].

Another fermentation option is the use of water kefir grains (WKG). WKGs are small, irregularly shaped, and translucent. In particular, WKGs are a symbiotic culture of microorganisms, specifically LAB and acetic acid bacteria (AAB), and yeasts found in a polysaccharide complex called α-glucan. In addition, the matrix can also contain levan [19]. The most prominent genera are *Lactobacillus*, *Acetobacter*, *Saccharomyces*, and other genera that occur simultaneously, but the exact composition is not yet defined. The presence of calcium and magnesium ions is important for the proper multiplication of grains. If these ions are not present, the grains are very small and more susceptible to microbial contamination. Therefore, it is recommended to use calcium-rich fruits or vegetables, including dried figs (162 mg/100 g), apricots (55 mg/100 g) or carrots (33 mg/100 g) [20].

In general, fermented whey beverages combined with fruit juices are primarily manufactured [21]. Therefore, the studies published so far have focused on the production of fermented beverages based on kefir starter cultures (milk or water kefir starter cultures) with the addition of mainly fruit components [22,23,24]. However, to date, there is little information in the available scientific literature on the development and characterization of vegetable beverages enriched with whey and fermented with milk or water kefir starter cultures. Furthermore, the application of water kefir culture for the manufacture of fermented beverages containing whey is scarce. The latter beverages may be suitable for a wide range of consumers due to their possible positive effects on humans. In particular, the enrichment of vegetable beverages with ingredients of dairy origin (i.e., the addition of whey) can enhance the functional properties of the aforementioned products.

Therefore, the present work was carried out with the objective of evaluating selected physicochemical, rheological, and sensory properties of a novel carrot juice whey-enriched beverage (carrot juice:whey ratios of 100:0; 95:5; 85:15; 75:25; 65:35) fermented with milk or water kefir starter cultures over a storage period of 21 days (at 4 ± 1 °C).

## 2. Materials and Methods

### 2.1. Materials

For the production of model fermented beverages, pasteurized carrot juice (dm-drogerie markt GmbH, Karlsruhe, Germany) was used, with a chemical composition of fat < 5.0% (*w*/*w*), carbohydrates 8.8% (*w*/*w*), and protein 1.0% (*w*/*w*). For whey powder (Mogador, s.r.o., Otrokovice, Czech Republic), the chemical composition was fat 0.5% (*w*/*w*), carbohydrate 76.0% (*w*/*w*), and protein 13.0% (*w*/*w*). Kefir cultures were used for fermentation: milk kefir starter culture (UNIBIOM s.r.o., Břeclav, Czech Republic), where the presence of microorganisms of the genera *Lactococcus*, *Leuconostoc*, *Lactobacillus*, *Saccharomyces*, and *Kluyveromyces* were found, and water kefir starter culture (UNIBIOM s.r.o., Czech Republic), where the presence of microorganisms of the genera *Lactobacillus*, *Lactrococcus*, *Leuconostoc*, *Acetobacter*, *Saccharomyces*, and *Kluyveromyces* were found (information obtained by the producer).

### 2.2. Manufacture of the Model Fermented Beverages

Model samples were produced by back-sloping. The inoculum for the production of model samples was prepared by adding 0.50 ± 0.03 g of lyophilized kefir culture in 500 mL of carrot juice. Inoculum fermentation was carried out at 20 ± 2 °C for 48 h (for the development of the active inoculum). Additionally, for the manufacture of fermented beverages, whey powder was reconstituted in distilled water (final concentration 5% *w*/*w*). Subsequently, the whey was thermally treated at 90 ± 1 °C for 10 ± 1 min and left to cool to 20 ± 2 °C. The samples were labeled as follows: M95_5 or W95_5 (95% *w*/*w* carrot juice and 5% *w*/*w* whey; M: sample fermented with milk kefir starter culture and W: sample fermented with water kefir starter culture), M85_15 or W85_15 (85% *w*/*w* carrot juice and 15% *w*/*w* whey), M75_25 or W75_25 (75% *w*/*w* carrot juice and 25% *w*/*w* whey), M65_35 or W65_35 (65% *w*/*w* carrot juice and 35% *w*/*w* whey). In addition, a sample consisting only of carrot juice served as a control sample (MCS, WCs). In all samples, 5% (*w*/*w*) of the active inoculum was added. After reaching the pH value of 4.3, the model samples were transferred to the refrigerator, where they were stored at 4 ± 1 °C. Analyses were subsequently performed after 1, 7, 14, and 21 days of storage. A total of 1500 mL of each fermented model beverage sample was prepared. Furthermore, 30 batches of model fermented beverages were manufactured: 5 variants of whey concentrations (including the control sample) × 2 types of starter culture × 3 repetitions.

### 2.3. Physicochemical Analysis

A Kern OTSS 45BE digital refractometer (Kern & Sohn GmbH, Balingen, Germany) was used to determine the total soluble solids (TSS; % *w*/*w*) of the samples. Total dissolved solids (TDS; ppt) were determined using a CyberScan CON 110 (Thermo Fisher Scientific Brno s.r.o., Brno, Czech Republic). Measurements of TSS and TDS were performed nine times (*n* = 9) at 20 ± 2 °C. Ethanol content (% *v*/*v*), density (kg·m^−3^), and the real degree of fermentation (RDF; % *w*/*w*) values were determined using an Anton Paar Alcolyzer Plus (Anton Paar GmbH, Graz, Austria) and an Anton Paar DMA 4500 density meter (Anton Paar GmbH, Austria). RDF was calculated directly by the Anton Paar Alcolyzer Plus software (version 2.2). Prior to the measurements, the samples were centrifuged and degassed using an EBA 21 centrifuge (Schoeller Instruments, s.r.o., Prague, Czech Republic, Germany) at 6000 rpm for 10 min. In addition, a water activity meter (AquaLab, Decagon Devices, Inc., Pullman, WA, USA) was used to determine the water activity values of the samples at 25 ± 1 °C. Before and during the measurement, the instrument was calibrated using a standard (a_w_ = 0.92 NaCl 2.33 mol in H_2_O; Qi Analytical, s.r.o., Prague, Czech Republic). The pH of the samples was determined (at 20 ± 1 °C) by inserting a glass tip electrode of a calibrated pH meter (Edge; Hanna Instruments Czech s.r.o., Prague, Czech Republic) directly into the samples. All analyses (with exception of TSS and TDS) were performed in triplicate (*n* = 3).

### 2.4. Rheological Analysis

The rheological analysis was determined with a HAAKE RheoStress 1 rheometer (Thermo Fisher Scientific, Waltham, MA, USA) equipped with a concentric cylinder geometry with a 2.1 mm gap. For each analysis, 1.0 mL of the sample tempered to 20.0 ± 0.1 °C was used. The samples were measured in sweep of shear rate (0–200 s^−1^) mode to calculate the steady-state rheological properties. Nonlinear regression analysis (Power Law model, Equation (1)) to process the flow curves obtained was used.

(1) $$\tau {=K\dot{\gamma }}^{n}$$

τ shear stress (Pa); K flow consistency index (Pa·s); $\dot{\gamma }$ hear rate (s^−1^); n Power Law index (dimensionless). The recorded values were the mean of at least six replicates (*n* = 6).

### 2.5. Instrumental Analysis of Color

A HunterLab UltraScan Pro Color spectrophotometer (Hunter Associates Laboratory, Inc., Reston, VA, USA) was used to determine the color of the samples. The CIE color scale (L*a*b*) with illumination D65 (normal daylight) and an angle of 10° was used for the evaluation. The parameter L* indicates lightness (luminosity) and takes values from 0 (black) to 100 (white). The parameter a* indicates the color spectrum from green (−) to red (+), the parameter b* indicates the color spectrum from blue (−) to yellow (+) [25]. The instrument was calibrated in reflectance mode excluding specular reflection using white (A41 1014-635 Rev. B; Hunterlab ColoTSSlex CZ; Hunter Associates Laboratory, Inc., Reston, VA, USA) and black (A41-1017-037 Rev A; Hunterlab ColoTSSlex CZ; Hunter Associates Laboratory, Inc., Reston, VA, USA) reference plates. Analyses were performed in triplicate (*n* = 3).

### 2.6. Turbidity Analysis

The determination of turbidity in the samples was performed using a TURB 430 IR portable turbidimeter (Xylem Analytics Germany Sales GmbH & Co. KG, WTW; Weilheim, Germany) equipped with an infrared LED lamp (860 nm), according to ISO 7027-1:2016 [26], using a nephelometric approach. Turbidity measurements are expressed in nephelometric turbidity units (NTU) [27]. Analyses were performed in triplicate (*n* = 3).

### 2.7. Sensory Analysis

The sensory parameters tested for the evaluation of the model samples were appearance, taste, aroma, and overall rating. A total of 12 assessors (9 women and 3 males) aged 22–51 years participated in the sensory evaluation. The samples were served in glass containers (50 mL, coded with three-digit codes) in a random order at a controlled temperature of 20 ± 2 °C. The sensory evaluation took place in a sensory analysis laboratory (separate sensory booths for each panelist under normal light conditions) equipped according to the standard ISO 8589, 2007 [28]. Water was served as a neutralizer. A 10-min pause was taken after each sample to prevent palate fatigue. Appearance, taste, and aroma were evaluated using a 5-point product quality scale (1—excellent, 3—good, 5—unacceptable; each point on the scale was objectively defined with quality parameters). For the overall rating, a 5-point scale was used, where 1—extraordinarily good and 5—extremely bad.

### 2.8. Statistical Analysis

The physicochemical and rheological parameters were compared by analysis of variance (one-factor ANOVA) and subsequent post-test (Tukey’s test) with 95% reliability. Data obtained were expressed as mean ± standard deviation. Additionally, the sensory properties of the model samples were verified by Kruskall–Wallis and Wilcoxon tests. The significance level used in the tests was 0.05. Statistical analyses were performed using Minitab^®^16 software (Minitab^®^, Ltd., Coventry, UK).

## 3. Results and Discussion

### 3.1. Physicochemical Analyses

The results of the physicochemical analyses of the model samples are presented in Table 1. The initial TSS values of the model samples with milk kefir starter culture ranged from 6.6 to 7.3 °Bx and with water kefir starter culture ranged from 6.1 to 7.1 °Bx. The initial TSS value decreased accordingly with the whey content of the developed samples (*p* < 0.05). During storage time, all TSS values decreased; the most significant reduction in TSS values was observed in control samples MCS and WCS. In contrast, the lowest decrease in TSS was observed in samples with 15 and 25% whey. According to Puerari et al. [25] and da Silva Araújo et al. [29], during fermentation, fermentable carbohydrates, including sucrose, glucose, and fructose, are consumed by the present microflora, generating other compounds, such as ethanol, CO_2_ and organic acids, which may explain the data obtained.

In the same token, the density of the model samples decreased during fermentation due to the conversion of fermentable carbohydrates to ethanol, CO_2_, and other sensory active substances, while the density decrease depended on the fermentation time [25]. The decrease was more pronounced on day 7 (*p* < 0.05), after that, the density was almost constant until day 21. The highest difference between the initial density values was observed in the control samples, while the lowest was observed in the samples with the addition of 15 and 25% whey. Moreover, the density of the samples also decreased during fermentation in the study of Paredes et al. [30], which dealt with the production of a fermented beverage from fruit and vegetable juices.

The limit of 0.5% (*v*/*v*) for non-alcoholic beverages is applied in most European countries [25]. According to the measured values, it can be concluded that only two samples with a milk kefir culture (M85_15, M75_25) and one sample with a water kefir culture (W85_15) can be classified as non-alcoholic (*p* < 0.05). The ethanol content of the samples with milk kefir starter culture was generally lower compared to that of the samples with water kefir starter culture. The final ethanol content ranged from 0.38 to 0.80% (*v*/*v*). Compared to samples with water kefir starter culture, the final ethanol content ranged from 0.5–0.91% *v*/*v*. Only the sample W85_15 was non-alcoholic on day 21 of the experiment; the other model samples with water kefir starter culture fell into the category of low-alcoholic beverages. These results can be compared to the study by Randazzo et al. [31]. Furthermore, according to Tzavaras et al. [32], the transition from milk to water kefir is indeed feasible, leading to the production of beverages with relatively higher ethanol content than milk kefir. Moreover, according to Beshkova et al. [33], the presence of ethanol is crucial for kefir-like products because it imparts the typical light alcoholic flavor and, along with CO_2_ primarily produced during yeast fermentation, gives the finished product the desired exotic notes and yeasty aroma.

The highest increase in the RDF value was observed on day 7 (*p* < 0.05), which corresponded to an increase in the ethanol content and a decrease in density. On the basis of the results obtained, it can be concluded that the activity of microorganisms was the highest during this period. The difference between the initial and final RDF values of the MCS and M65_35 model samples was the highest (*p* < 0.05). These samples, by their composition, represented the most suitable medium for fermentation and the microorganisms that were part of the kefir culture. However, the lowest RDF value was observed in samples M85_15 and M75_25. In general, the RDF value was higher in the samples with water kefir, which could be due to the more suitable medium for fermentation given by the water kefir culture.

Model samples showed an increase in the TDS value during 21 days of storage in all cases (*p* < 0.05). The conductivity of the model samples could be explained by the recorded TDS values; a greater value indicated more electrolytes or dissolved solids, which were present in the fermented beverages [34].

The water activity during storage decreased slightly in the model samples; however, the differences between the initial and final values were not statistically significant (*p* > 0.05).

The initial pH of the samples (determined immediately after manufacture) was 5.79 ± 0.01 (0 day) and 5.81 ± 0.02 (0 day) for samples manufactured with milk kefir starter culture and water kefir starter culture, respectively. The pH values of the model fermented beverages were (1 day) 4.42 ± 0.02 (for samples manufactured with milk kefir starter culture) and 4.28 ± 0.02 (for samples manufactured with water kefir starter culture), respectively. In particular, this decrease in the pH values is the result of organic acid production by LAB and yeasts. The results agree with those of M’hir et al. [35]. The prolongation of the storage time slightly affected the pH values of the tested samples (*p* > 0.05). The pH values of the samples on day 21 were 4.05 ± 0.01 (for samples manufactured with milk kefir starter culture) and 4.02 ± 0.01 (for samples manufactured with water kefir starter culture), regardless of the applied whey concentration. Although there was a decrease in the pH values of the samples during storage time, this decrease was not significant (*p* > 0.05). In general, acidic pH is important to preserve kefir-like beverages from spoilage microorganisms [35].

### 3.2. Rheological Analysis

**Figure 1:**
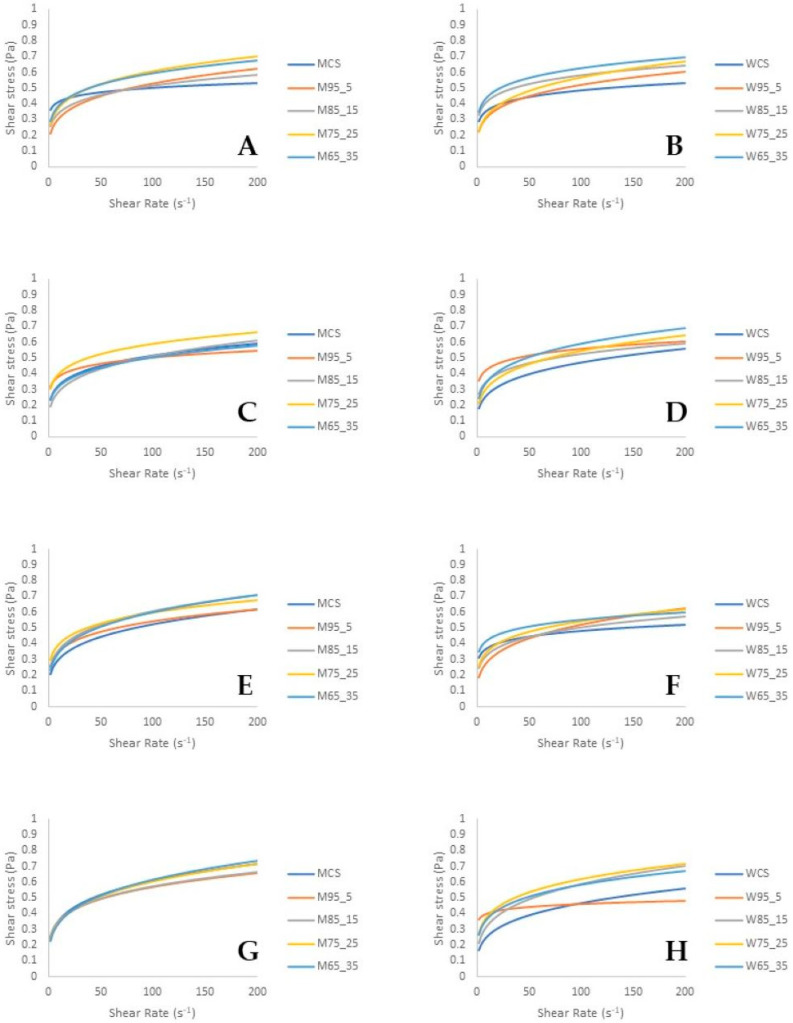
Flow curves of the model samples during a period of 21 days; samples with milk kefir starter culture day 1 (part **A**), day 7 (part **C**), day 14 (part **E**), day 21 (part **G**); model samples with water kefir starter culture day 1 (part **B**), day 7 (part **D**), day 14 (part **F**), day 21 (part **H**). MCS and WCS: control samples (M: sample fermented with milk kefir starter culture and W: sample fermented with water kefir starter culture), M95_5 or W95_5 (95% *w*/*w* carrot juice and 5% *w*/*w* whey), M85_15 or W85_15 (85% *w*/*w* carrot juice and 15% *w*/*w* whey), M75_25 or W75_25 (75% *w*/*w* carrot juice and 25% *w*/*w* whey), M65_35 or W65_35 (65% *w*/*w* carrot juice and 35% *w*/*w* whey).

In general, there were changes in the flow curves of the model samples during the 21-day experiment period (Figure 1). In particular, as can be seen in Figure 1, with an increase in shear rate, shear stress increases. On the first day of the experiment, WCS exhibited lower shear stress/shear rate values compared to the samples with whey addition, indicating a potential influence of the whey content on the flow properties of the samples. However, this trend was not observed in samples with milk kefir cultures. The flow curves of the model samples with milk kefir culture were relatively similar during storage; however, statistically significant differences were observed between the individual samples (*p* < 0.05). In addition, the differences in the flow curves of the model samples with the water kefir culture were more pronounced.

The Power Law model exhibited an adjustment to the rheological data, as evidenced by the coefficients of determination (R^2^) that exceeded 0.99 (Table 2). During the experiment, the flow properties of the model samples changed significantly (*p* < 0.05). The addition of whey affected the values of K and n, and differences were observed at the same time between the sample concentrations. All fermented beverage samples (regardless of the whey concentration used or starter culture applied) were discovered to exhibit non-Newtonian fluid behavior (shear thinning or pseudoplastic) and the structures were characterized by relatively low resistance to flow. Fermented carrot juice beverages enriched with whey were reported to have a pseudoplastic fluid character because the n values were less than 1. Pseudoplastic behavior (shear thinning character) occurs as a result of the break-down of structural units (hydrocolloids) because of the hydrodynamic forces generated during the shearing process [36]. Similarly, in rheological studies on kefir, which is a type of fermented beverage, the flow curves showed that it has shear-thinning (pseudoplastic) properties [37]. Furthermore, after increasing during the first seven days of the storage period, the consistency index of most samples decreased (Table 2; *p* < 0.05) [38,39,40,41]. The results are in accordance with those previously reported by Oliveira et al. [42] and Amaral et al. [43].

### 3.3. Instrumental Color Analysis and Turbidity Analysis

Colorimetric parameters (Table 3) L*, a*, and b* showed significant differences (*p* < 0.05) between samples as a function of increasing whey addition. On the contrary, the differences between the use of milk and water kefir cultures were not significant (*p* > 0.05). In general, the addition of whey affected all colorimetric parameters. The L* values of all model samples were close to 0; however, the values increased as the whey content increased. Both a* and b* parameters took positive values; therefore, the color of the model samples can be described as yellow with a red tint.

Table 3 shows the results of the turbidity values. As the whey content of the samples increased, the turbidity values decreased, so the samples MCS and WCS, which contained only carrot juice, showed the highest turbidity values. These results are in accordance with those previously reported in the study of Liao et al. [44].

### 3.4. Sensory Analysis

The results of the sensory analysis are shown in Table 4. The model samples were evaluated for appearance, taste, aroma, and overall rating. During fermentation, there may be a change in appearance due to oxidation, an increase in acidity, and a consequent change in taste and aroma, which might affect the overall impression of the tested samples [45]. Almost all model samples showed changes in organoleptic properties during storage; however, there were no differences between model samples (*p* > 0.05) in the appearance assessment, and all samples were judged to have a satisfactory appearance. In contrast, during the prolonged storage time, the taste and aroma deteriorated in most of the model samples (*p* < 0.05). In terms of overall rating, the samples M75_25 and W75_25 containing 25% whey were the best rated during the 21-day storage experiment.

## 4. Conclusions

The purpose of this work was to evaluate the selected physicochemical, rheological, and sensory properties of a new whey-enriched carrot juice beverage (carrot juice: whey ratios of 100:0; 95:5; 85:15; 75:25; 65:35) fermented with milk or water kefir starter cultures over a storage period of 21 days (at 4 ± 1 °C). During the experiment, changes in physicochemical properties were observed, such as a decrease in density, TSS, pH, ethanol content, RDF, and TDS. In addition, all evaluated samples (regardless of the whey concentration used or starter culture applied) were discovered to exhibit non-Newtonian fluid behavior (shear thinning or pseudoplastic), and the structures were characterized by relatively low resistance to flow. At the same time, changes in the organoleptic properties of the model samples were also monitored. Samples containing 25% (% *w*/*w*) of whey were considered the most acceptable samples, regardless of the starter culture used. Our findings demonstrated the potential of whey as an ingredient in the formulation of functional food products, particularly fermented beverages. Although whey is already used in the formulation of some beverages, our work further highlighted its potential for use in vegetable-based fermented beverages. Thus, the use of whey as a key ingredient in the production of functional foods could lead to a sustainable economy, as the disposal of whey can cause serious environmental issues. Further research can explore the antioxidant profile, antioxidant ability, microbiological characteristics, and commercial potential of the products mentioned above.

## Figures and Tables

**Table 1:** Physicochemical parameters of the evaluated carrot juice whey-enriched beverages fermented with milk and water kefir starter cultures.

Sample *	Time (Days)	TSS ^1^ (°Bx)	Density (kg·m^−3^)	Ethanol (% *v*/*v*)	RDF ^1^ (% *w*/*w*)	TDS ^1^ (ppt)	a_w_
MCS		7.3 ^aA^ ± 0.2	1.031 ^aA^ ± 0.001	0.12 ^aA^ ± 0.01	2.21 ^aA^ ± 0.02	6.88 ^aA^ ± 0.09	0.992 ^aA^ ± 0.001
M95_5		7.2 ^aA^ ± 0.1	1.031 ^aA^ ± 0.001	0.15 ^bA^ ± 0.02	2.98 ^bA^ ± 0.01	6.53 ^bA^ ± 0.02	0.993 ^aA^ ± 0.002
M85_15		7.1 ^aA^ ± 0.1	1.030 ^aA^ ± 0.002	0.21 ^cA^ ± 0.01	3.29 ^cA^ ± 0.03	6.47 ^cA^ ± 0.04	0.992 ^aA^ ± 0.001
M75_25		6.7 ^bA^ ± 0.1	1.028 ^aA^ ± 0.001	0.25 ^dA^ ± 0.01	3.95 ^dA^ ± 0.01	6.07 ^dA^ ± 0.09	0.993 ^aA^ ± 0.001
M65_35		6.6 ^bA^ ± 0.2	1.027 ^aA^ ± 0.001	0.24 ^dA^ ± 0.02	4.98 ^eA^ ± 0.01	6.13 ^dA^ ± 0.07	0.991 ^aA^ ± 0.001
WCS	1	6.9 ^aB^ ± 0.3	1.028 ^aA^ ± 0.002	0.48 ^aB^ ± 0.02	9.04 ^aB^ ± 0.02	6.61 ^aB^ ± 0.08	0.993 ^aA^ ± 0.002
W95_5		7.1 ^aA^ ± 0.2	1.028 ^aB^ ± 0.001	0.38 ^bB^ ± 0.01	7.23 ^bB^ ± 0.01	6.57 ^aA^ ± 0.06	0.992 ^aA^ ± 0.001
W85_15		6.5 ^aB^ ± 0.1	1.027 ^aA^ ± 0.002	0.46 ^cB^ ± 0.01	8.87 ^cB^ ± 0.01	6.33 ^bB^ ± 0.04	0.993 ^aA^ ± 0.001
W75_25		6.2 ^bB^ ± 0.1	1.025 ^aA^ ± 0.002	0.45 ^cB^ ± 0.02	9.51 ^dB^ ± 0.02	5.88 ^cB^ ± 0.07	0.993 ^aA^ ± 0.002
W65_35		6.1 ^bB^ ± 0.1	1.024 ^aB^ ± 0.001	0.61 ^dB^ ± 0.01	11.46 ^eB^ ± 0.03	6.06 ^dA^ ± 0.01	0.992 ^aA^ ± 0.001
MCS	7	5.6 ^aA^ ± 0.1	1.026 ^aA^ ± 0.001	0.77 ^aA^ ± 0.01	14.45 ^aA^ ± 0.02	9.01 ^aA^ ± 0.04	0.991 ^aA^ ± 0.001
M95_5		6.1 ^bA^ ± 0.2	1.028 ^aA^ ± 0.001	0.48 ^bA^ ± 0.02	9.09 ^bA^ ± 0.03	9.01 ^aA^ ± 0.02	0.991 ^aA^ ± 0.001
M85_15		5.9 ^bA^ ± 0.2	1.028 ^aA^ ± 0.001	0.32 ^cA^ ± 0.01	6.28 ^cA^ ± 0.01	8.92 ^bA^ ± 0.08	0.991 ^aA^ ± 0.001
M75_25		5.5 ^aA^ ± 0.1	1.026 ^aA^ ± 0.002	0.33 ^cA^ ± 0.01	6.74 ^dA^ ± 0.01	8.44 ^cA^ ± 0.05	0.991 ^aA^ ± 0.002
M65_35		5.3 ^aA^ ± 0.2	1.024 ^aA^ ± 0.001	0.42 ^dA^ ± 0.01	8.95 ^eA^ ± 0.02	8.01 ^dA^ ± 0.05	0.991 ^aA^ ± 0.001
WCS		5.6 ^aA^ ± 0.1	1.025 ^aA^ ± 0.001	0.96 ^aB^ ± 0.01	17.85 ^aB^ ± 0.02	9.71 ^aB^ ± 0.06	0.991 ^aA^ ± 0.001
W95_5		6.1 ^bA^ ± 0.1	1.026 ^aA^ ± 0.001	0.75 ^bB^ ± 0.02	14.16 ^bB^ ± 0.01	9.57 ^bB^ ± 0.01	0.991 ^aA^ ± 0.001
W85_15		5.7 ^aA^ ± 0.2	1.026 ^aA^ ± 0.003	0.61 ^cB^ ± 0.01	11.79 ^cB^ ± 0.02	9.26 ^vB^ ± 0.09	0.991 ^aA^ ± 0.002
W75_25		5.4 ^aA^ ± 0.1	1.024 ^aA^ ± 0.002	0.64 ^cB^ ± 0.02	13.13 ^dB^ ± 0.02	9.26 ^vB^ ± 0.03	0.991 ^aA^ ± 0.001
W65_35		4.4 ^cB^ ± 0.2	1.022 ^aA^ ± 0.002	0.77 ^bB^ ± 0.02	16.53 ^eB^ ± 0.01	9.03 ^dB^ ± 0.04	0.992 ^aA^ ± 0.002
MCS	14	5.7 ^aA^ ± 0.1	1.025 ^aA^ ± 0.001	0.83 ^aA^ ± 0.01	15.06 ^aA^ ± 0.01	9.47 ^aA^ ± 0.09	0.989 ^aA^ ± 0.001
M95_5		6.1 ^bA^ ± 0.2	1.027 ^aA^ ± 0.001	0.56 ^bA^ ± 0.02	10.71 ^bA^ ± 0.04	9.49 ^aA^ ± 0.07	0.991 ^aA^ ± 0.002
M85_15		6.1 ^bA^ ± 0.2	1.027 ^aA^ ± 0.001	0.33 ^cA^ ± 0.01	6.52 ^cA^ ± 0.02	9.14 ^bA^ ± 0.01	0.990 ^aA^ ± 0.001
M75_25		5.6 ^aA^ ± 0.1	1.026 ^aA^ ± 0.001	0.34 ^cA^ ± 0.02	6.97 ^dA^ ± 0.02	8.46 ^aA^ ± 0.05	0.990 ^aA^ ± 0.002
M65_35		5.5 ^aA^ ± 0.1	1.024 ^aA^ ± 0.002	0.48 ^dA^ ± 0.02	10.28 ^eA^ ± 0.01	8.10 ^cA^ ± 0.02	0.990 ^aA^ ± 0.002
WCS		5.7 ^aA^ ± 0.1	1.025 ^aA^ ± 0.002	0.91 ^aB^ ± 0.02	16.99 ^aB^ ± 0.01	9.57 ^aA^ ± 0.05	0.989 ^aA^ ± 0.001
W95_5		5.8 ^aA^ ± 0.1	1.026 ^aA^ ± 0.001	0.75 ^bB^ ± 0.02	13.69 ^bB^ ± 0.02	9.57 ^aA^ ± 0.07	0.991 ^aA^ ± 0.002
W85_15		5.9 ^aA^ ± 0.2	1.025 ^aA^ ± 0.002	0.58 ^cB^ ± 0.01	11.48 ^cB^ ± 0.02	8.88 ^bA^ ± 0.08	0.991 ^aA^ ± 0.001
W75_25		5.6 ^aA^ ± 0.1	1.024 ^aA^ ± 0.001	0.66 ^dB^ ± 0.01	13.21 ^dB^ ± 0.01	8.91 ^cB^ ± 0.07	0.992 ^aA^ ± 0.002
W65_35		4.9 ^bB^ ± 0.1	1.021 ^bA^ ± 0.002	0.77 ^bB^ ± 0.02	16.64 ^eB^ ± 0.03	8.57 ^dB^ ± 0.12	0.992 ^aA^ ± 0.002
MCS	21	6.2 ^aA^ ± 0.1	1.025 ^aA^ ± 0.002	0.81 ^aA^ ± 0.02	15.25 ^aA^ ± 0.01	9.54 ^aA^ ± 0.02	0.989 ^aA^ ± 0.002
M95_5		6.3 ^aA^ ± 0.1	1.026 ^aA^ ± 0.001	0.68 ^bA^ ± 0.01	12.95 ^bA^ ± 0.03	9.79 ^bA^ ± 0.07	0.991 ^aA^ ± 0.001
M85_15		6.5 ^bA^ ± 0.1	1.027 ^aA^ ± 0.001	0.38 ^cA^ ± 0.01	7.49 ^cA^ ± 0.01	9.52 ^aA^ ± 0.04	0.991 ^aA^ ± 0.001
M75_25		6.1 ^aA^ ± 0.2	1.026 ^aA^ ± 0.002	0.39 ^cA^ ± 0.02	7.97 ^dA^ ± 0.02	9.04 ^cA^ ± 0.06	0.091 ^aA^ ± 0.001
M65_35		5.7 ^cA^ ± 0.1	1.023 ^aA^ ± 0.001	0.78 ^dA^ ± 0.02	11.21 ^eA^ ± 0.01	8.58 ^aA^ ± 0.05	0.991 ^aA^ ± 0.001
WCS		5.9 ^aA^ ± 0.3	1.025 ^aA^ ± 0.002	0.91 ^aB^ ± 0.01	17.14 ^aB^ ± 0.03	8.88 ^aB^ ± 0.05	0.988 ^aA^ ± 0.002
W95_5		6.3 ^aA^ ± 0.1	1.026 ^aA^ ± 0.001	0.72 ^bB^ ± 0.01	14.01 ^bB^ ± 0.01	8.83 ^aB^ ± 0.03	0.991 ^aA^ ± 0.001
W85_15		6.1 ^aB^ ± 0.1	1.025 ^aA^ ± 0.002	0.51 ^cB^ ± 0.02	10.21 ^cB^ ± 0.01	8.76 ^aB^ ± 0.04	0.989 ^aA^ ± 0.001
W75_25		6.1 ^aA^ ± 0.1	1.024 ^aA^ ± 0.002	0.66 ^dB^ ± 0.02	13.87 ^dB^ ± 0.02	8.84 ^aB^ ± 0.09	0.989 ^aA^ ± 0.002
W65_35		5.1 ^bB^ ± 0.1	1.021 ^aA^ ± 0.001	0.78 ^eA^ ± 0.01	16.93 ^eB^ ± 0.01	8.51 ^bB^ ± 0.05	0.991 ^aA^ ± 0.002

^1^ TSS: Total Soluble Solids, ^1^ TDS: Total Dissolved Solids ^1^ RDF: Real Degree of Fermentation. Results are expressed as mean value ± standard deviation; * MCS and WCS: control samples (M: sample fermented with milk kefir starter culture and W: sample fermented with water kefir starter culture), M95_5 or W95_5 (95% *w*/*w* carrot juice and 5% *w*/*w* whey), M85_15 or W85_15 (85% *w*/*w* carrot juice and 15% *w*/*w* whey), M75_25 or W75_25 (75% *w*/*w* carrot juice and 25% *w*/*w* whey), M65_35 or W65_35 (65% *w*/*w* carrot juice and 35% *w*/*w* whey). Mean values within a column (difference between whey concentration, comparing the same starter culture type; the control samples were also evaluated) followed by different superscript letters differ (*p* < 0.05); the samples stored for different times were evaluated independently. Mean values within a column (difference between starter culture type; comparing the same whey concentration; the control samples were also evaluated) followed by different uppercase letters statistically differ (*p* < 0.05); the samples stored for different times were evaluated independently.

**Table 2:** Rheological parameters (K—flow consistency index, n—Power Law index, R^2^—coefficient of determination) of the model samples obtained from the Power Law model.

Sample *	Time (Days)	K (Pa·s)	n	R^2^
MCS	1	0.341 ^aA^ ± 0.004	0.085 ^aA^ ± 0.002	0.9957
M95_5		0.177 ^bA^ ± 0.002	0.238 ^bA^ ± 0.001	0.9969
M85_15		0.225 ^cA^ ± 0.003	0.211 ^cA^ ± 0.002	0.9955
M75_25		0.231 ^dA^ ± 0.005	0.211 ^cA^ ± 0.004	0.9918
M65_35		0.257 ^eA^ ± 0.001	0.183 ^dA^ ± 0.002	0.9983
WCS		0.266 ^aB^ ± 0.002	0.131 ^aB^ ± 0.002	0.9975
W95_5		0.195 ^bB^ ± 0.003	0.214 ^bB^ ± 0.001	0.9981
W85_15		0.299 ^cB^ ± 0.002	0.144 ^cB^ ± 0.002	0.9983
W75_25		0.191 ^dB^ ± 0.001	0.237 ^dB^ ± 0.003	0.9972
W65_35		0.311 ^eB^ ± 0.001	0.152 ^eB^ ± 0.002	0.9986
MCS	7	0.205 ^aA^ ± 0.004	0.199 ^aA^ ± 0.001	0.9999
M95_5		0.293 ^bA^ ± 0.002	0.116 ^bA^ ± 0.003	0.9941
M85_15		0.159 ^cA^ ± 0.002	0.253 ^cA^ ± 0.004	0.9952
M75_25		0.271 ^dA^ ± 0.003	0.168 ^dA^ ± 0.002	0.9992
M65_35		0.202 ^aA^ ± 0.006 ^b^	0.199 ^aA^ ± 0.002	0.9967
WCS		0.151 ^aB^ ± 0.003	0.248 ^aB^ ± 0.002	0.9993
W95_5		0.325 ^bB^ ± 0.001	0.117 ^bA^ ± 0.002	0.9978
W85_15		0.239 ^cB^ ± 0.001	0.171 ^cB^ ± 0.001	0.9958
W75_25		0.181 ^dB^ ± 0.002	0.241 ^aB^ ± 0.004	0.9987
W65_35		0.211 ^eB^ ± 0.004	0.223 ^dB^ ± 0.002	0.9989
MCS	14	0.172 ^aA^ ± 0.002	0.242 ^aA^ ± 0.001	0.9989
M95_5		0.229 ^bA^ ± 0.001	0.187 ^bA^ ± 0.004	0.9976
M85_15		0.212 ^cA^ ± 0.003	0.228 ^cA^ ± 0.002	0.9967
M75_25		0.259 ^dA^ ± 0.002	0.181 ^bA^ ± 0.003	0.9967
M65_35		0.198 ^eA^ ± 0.004	0.241 ^aA^ ± 0.002	0.9929
WCS		0.285 ^aB^ ± 0.002	0.113 ^aB^ ± 0.002	0.9999
W95_5		0.154 ^bB^ ± 0.003	0.264 ^bB^ ± 0.001	0.9976
W85_15		0.213 ^cA^ ± 0.002	0.186 ^cB^ ± 0.004	0.9967
W75_25		0.229 ^dB^ ± 0.004	0.187 ^cA^ ± 0.003	0.9967
W65_35		0.321 ^eB^ ± 0.001	0.118 ^dB^ ± 0.001	0.9929
MCS	21	0.208 ^aA^ ± 0.003	0.228 ^aA^ ± 0.001	0.9953
M95_5		0.219 ^bA^ ± 0.002	0.107 ^bA^ ± 0.003	0.9955
M85_15		0.215 ^cA^ ± 0.001	0.213 ^cA^ ± 0.002	0.9956
M75_25		0.193 ^dA^ ± 0.007	0.247 ^dA^ ± 0.005	0.9949
M65_35		0.187 ^eA^ ± 0.004	0.259 ^eA^ ± 0.004	0.9939
WCS		0.141 ^aB^ ± 0.002	0.262 ^aB^ ± 0.004	0.9991
W95_5		0.349 ^bB^ ± 0.003	0.061 ^bB^ ± 0.002	0.9955
W85_15		0.176 ^cB^ ± 0.001	0.261 ^aB^ ± 0.001	0.9956
W75_25		0.134 ^dB^ ± 0.003	0.211 ^cB^ ± 0.003	0.9949
W65_35		0.233 ^eB^ ± 0.002	0.201 ^eB^ ± 0.001	0.9939

Results are expressed as mean value ± standard deviation. * MCS and WCS: control samples (M: sample fermented with milk kefir starter culture and W: sample fermented with water kefir starter culture), M95_5 or W95_5 (95% *w*/*w* carrot juice and 5% *w*/*w* whey), M85_15 or W85_15 (85% *w*/*w* carrot juice and 15% *w*/*w* whey), M75_25 or W75_25 (75% *w*/*w* carrot juice and 25% *w*/*w* whey), M65_35 or W65_35 (65% *w*/*w* carrot juice and 35% *w*/*w* whey). Mean values within a column (difference between whey concentration, comparing the same starter culture type; the control samples were also evaluated) followed by different superscript letters differ (*p* < 0.05); the samples stored for different times were evaluated independently. Mean values within a column (difference between starter culture type; comparing the same whey concentration; the control samples were also evaluated) followed by different uppercase letters statistically differ (*p* < 0.05); the samples stored for different times were evaluated independently.

**Table 3:** Colorimetric parameters (L* lightness; a* from green (−) to red (+); b* from blue (−) to yellow (+)) and turbidity results of the model samples.

Samples ^1^	Colorimetric Parameters			Turbidity (NTU)
	L*	a*	b*	
MCS	15.39 ^aA^ ± 0.03	35.43 ^aA^ ± 0.03	26.53 ^aA^ ± 0.06	885 ^aA^ ± 2
M95_5	16.83 ^bA^ ± 0.03	36.30 ^bA^ ± 0.03	29.02 ^bA^ ± 0.06	685 ^bA^ ± 1
M85_15	18.86 ^cA^ ± 0.09	37.13 ^cA^ ± 0.07	32.51 ^cA^ ± 0.16	465 ^cA^ ± 1
M75_25	21.35 ^dA^ ± 0.06	38.06 ^dA^ ± 0.04	36.81 ^dA^ ± 0.10	455 ^dA^ ± 1
M65_35	24.91 ^eA^ ± 0.06	39.07 ^eA^ ± 0.04	42.96 ^eA^ ± 0.11	451 ^eA^ ± 1
WCS	15.41 ^aA^ ± 0.02	35.44 ^aA^ ± 0.02	26.56 ^aA^ ± 0.03	882 ^aA^ ± 1
W95_5	16.82 ^bA^ ± 0.04	36.29 ^bA^ ± 0.03	29.01 ^bA^ ± 0.06	679 ^bA^ ± 1
W85_15	18.91 ^cA^ ± 0.04	37.17 ^cA^ ± 0.02	32.58 ^cA^ ± 0.07	463 ^cA^ ± 2
W75_25	21.35 ^dA^ ± 0.03	38.06 ^dA^ ± 0.02	36.82 ^dA^ ± 0.05	452 ^dA^ ± 2
W65_35	24.94 ^eA^ ± 0.01	39.08 ^eA^ ± 0.01	43.01 ^eA^ ± 0.02	449 ^eA^ ± 2

Results are expressed as mean value ± standard deviation. ^1^ MCS and WCS: control samples (M: sample fermented with milk kefir starter culture and W: sample fermented with water kefir starter culture), M95_5 or W95_5 (95% *w*/*w* carrot juice and 5% *w*/*w* whey), M85_15 or W85_15 (85% *w*/*w* carrot juice and 15% *w*/*w* whey), M75_25 or W75_25 (75% *w*/*w* carrot juice and 25% *w*/*w* whey), M65_35 or W65_35 (65% *w*/*w* carrot juice and 35% *w*/*w* whey). Mean values within a column (difference between whey concentration, comparing the same starter culture type; the control samples were also evaluated) followed by different superscript letters differ (*p* < 0.05). Mean values within a column (difference between starter culture type; comparing the same whey concentration; the control samples were also evaluated) followed by different uppercase letters statistically differ (*p* < 0.05).

**Table 4:** Results of the sensory analysis of the model samples (Appearance, Aroma, Taste, Overall Rating).

Sample *	Time (Days)	Parameters			
		Appearance ^1^	Taste ^1^	Aroma ^1^	Overall Rating ^1^
MCS	1	2 ^aA^	2 ^aA^	1 ^aA^	2 ^aA^
M95_5		2 ^aA^	2 ^aA^	1 ^aA^	2 ^aA^
M85_15		2 ^aA^	2 ^aA^	1 ^aA^	2 ^aA^
M75_25		2 ^aA^	1 ^bA^	1 ^aA^	1 ^bA^
M65_35		2 ^aA^	2 ^aA^	1 ^aA^	2 ^aA^
WCS		2 ^aA^	2 ^aA^	1 ^aA^	2 ^aA^
W95_5		2 ^aA^	2 ^aA^	1 ^aA^	2 ^aA^
W85_15		2 ^aA^	1 ^bB^	1 ^aA^	1 ^bB^
W75_25		2 ^aA^	1 ^bA^	1 ^aA^	1 ^bA^
W65_35		2 ^aA^	2 ^aA^	1 ^aA^	2 ^aA^
MCS	7	2 ^aA^	3 ^aA^	2 ^aA^	3 ^aA^
M95_5		2 ^aA^	3 ^aA^	2 ^aA^	3 ^aA^
M85_15		2 ^aA^	2 ^bA^	2 ^aA^	2 ^bA^
M75_25		2 ^aA^	1 ^cA^	1 ^bA^	1 ^cA^
M65_35		2 ^aA^	3 ^aA^	2 ^aA^	3 ^aA^
WCS		2 ^aA^	2 ^aB^	1 ^aB^	2 ^aB^
W95_5		2 ^aA^	3 ^bA^	2 ^bA^	2 ^aB^
W85_15		2 ^aA^	2 ^aA^	1 ^aB^	2 ^aA^
W75_25		1 ^bB^	1 ^cA^	1 ^aA^	1 ^bA^
W65_35		2 ^aA^	2 ^aB^	2 ^bA^	2 ^aB^
MCS	14	2 ^aA^	3 ^aA^	3 ^aA^	3 ^aA^
M95_5		2 ^aA^	3 ^aA^	3 ^aA^	3 ^aA^
M85_15		2 ^aA^	3 ^aA^	2 ^bA^	2 ^bA^
M75_25		2 ^aA^	1 ^bA^	1 ^cA^	1 ^cA^
M65_35		2 ^aA^	3 ^aA^	2 ^bA^	3 ^aA^
WCS		2 ^aA^	3 ^aA^	3 ^aA^	3 ^aA^
W95_5		2 ^aA^	3 ^aA^	2 ^bB^	2 ^bB^
W85_15		2 ^aA^	2 ^bB^	1 ^cB^	2 ^bA^
W75_25		2 ^aA^	2 ^bB^	1 ^cA^	1 ^cA^
W65_35		2 ^aA^	2 ^bB^	2 ^bA^	2 ^bB^
MCS	21	2 ^aA^	3 ^aA^	3 ^aA^	3 ^aA^
M95_5		2 ^aA^	3 ^aA^	3 ^aA^	3 ^aA^
M85_15		2 ^aA^	3 ^aA^	2 ^bA^	3 ^aA^
M75_25		2 ^aA^	1 ^bA^	1 ^cA^	1 ^bA^
M65_35		2 ^aA^	3 ^aA^	3 ^aA^	3 ^aA^
WCS		2 ^aA^	3 ^aA^	3 ^aA^	3 ^aA^
W95_5		2 ^aA^	3 ^aA^	2 ^bB^	2 ^bB^
W85_15		2 ^aA^	3 ^aA^	2 ^bA^	2 ^bB^
W75_25		2 ^aA^	2 ^bB^	1 ^cA^	1 ^cA^
W65_35		2 ^aA^	3 ^aA^	3 ^aA^	3 ^aA^

* MCS and WCS: control samples (M: sample fermented with milk kefir starter culture and W: sample fermented with water kefir starter culture), M95_5 or W95_5 (95% *w*/*w* carrot juice and 5% *w*/*w* whey), M85_15 or W85_15 (85% *w*/*w* carrot juice and 15% *w*/*w* whey), M75_25 or W75_25 (75% *w*/*w* carrot juice and 25% *w*/*w* whey), M65_35 or W65_35 (65% *w*/*w* carrot juice and 35% *w*/*w* whey). Results are reported as medians. Median values within a column (difference between whey concentration, comparing the same starter culture type; the control samples were also evaluated) followed by different superscript letters differ (*p* < 0.05); the samples stored for different times were evaluated independently. Median values within a column (difference between starter culture type; comparing the same whey concentration; the control samples were also evaluated) followed by different uppercase letters statistically differ (*p* < 0.05); the samples stored for different times were evaluated independently. ^1^ Appearance: 1—excellent, 3—good, 5—unacceptable. Taste: 1—excellent, 3—good, 5—unacceptable. Aroma: 1—excellent, 3—good, 5—unacceptable. Overall rating: 1—extraordinarily good; 5—extremely bad.

## Data Availability

The data used to support the findings of this study can be made available by the corresponding author upon request.
